# When Foot Drop Tells a Bigger Story: POEMS (Polyneuropathy, Organomegaly, Endocrinopathy, Monoclonal Plasma Cell Disorder, and Skin Changes) Syndrome Revealed by Femoral Plasmacytoma

**DOI:** 10.7759/cureus.103751

**Published:** 2026-02-16

**Authors:** Usamah Al-Anbagi, Safwan Ahmed, Hussam Telfah, Mohamed Mohamedali, Abdulqadir J Nashwan, Mohammed Ahmed

**Affiliations:** 1 Department of Internal Medicine, Hazm Mebaireek General Hospital, Hamad Medical Corporation, Doha, QAT; 2 Department of Neurology, Hamad Medical Corporation, Doha, QAT; 3 Department of Laboratory Medicine and Pathology, Hamad Medical Corporation, Doha, QAT; 4 Department of Internal Medicine, Hamad Medical Corporation, Doha, QAT; 5 Department of Nursing and Midwifery Research, Hamad Medical Corporation, Doha, QAT

**Keywords:** case report, lambda light chain, plasmacytoma, poems syndrome, polyneuropathy, thrombocytosis

## Abstract

POEMS (polyneuropathy, organomegaly, endocrinopathy, monoclonal plasma cell disorder, and skin changes) syndrome is a rare multisystem disorder caused by a monoclonal plasma cell dyscrasia, often presenting with a constellation of neuropathy, organomegaly, endocrinopathy, monoclonal protein, and skin changes. Its initial manifestations frequently overlap with more common causes of chronic progressive neuropathy, such as chronic inflammatory demyelinating polyneuropathy (CIDP) and diabetic or paraproteinemic neuropathies, which can obscure the diagnosis and delay definitive treatment. We report a 45-year-old male with progressive bilateral lower-limb distal-predominant weakness and numbness over three months, in whom the clinical picture and cerebrospinal fluid findings initially supported a working diagnosis of CIDP, despite coexisting thrombocytosis, skin hyperpigmentation, and newly diagnosed diabetes mellitus. Electroneuromyography showed severe sensorimotor mixed axonal and demyelinating neuropathy, and cerebrospinal fluid analysis demonstrated albuminocytologic dissociation, further reinforcing the initial impression of CIDP and illustrating the diagnostic conflict. However, serum studies identified an IgA lambda monoclonal protein, and fluorodeoxyglucose positron emission tomography revealed a solitary, metabolically active lesion in the right femur, raising suspicion for an underlying plasma cell dyscrasia rather than isolated inflammatory neuropathy. Histopathological examination of the femoral lesion confirmed a lambda-restricted plasmacytoma with minimal bone marrow plasma cell infiltration, fulfilling the diagnostic criteria for POEMS syndrome in conjunction with polyneuropathy, monoclonal plasma cell disorder, plasmacytoma, thrombocytosis, and skin changes. This case highlights the importance of considering POEMS syndrome in patients with atypical or treatment-refractory CIDP-like presentations and systemic features, and it underscores how a stepwise, multidisciplinary diagnostic approach incorporating electrophysiology, advanced imaging, and targeted histopathology can resolve overlapping differentials and guide timely, targeted therapy

## Introduction

POEMS syndrome is a rare paraneoplastic syndrome driven by an underlying plasma cell dyscrasia. The acronym POEMS represents its cardinal features: polyneuropathy, organomegaly, endocrinopathy, monoclonal plasma cell disorder, and skin changes [1,2]. However, the clinical spectrum is broader and may include sclerotic bone lesions, papilledema, extravascular volume overload, and hematologic abnormalities such as thrombocytosis or polycythemia [1-5]. Because these manifestations evolve gradually and are often incomplete at presentation, POEMS syndrome is frequently misdiagnosed or attributed to more common causes of neuropathy, such as chronic inflammatory demyelinating polyneuropathy (CIDP) or diabetic neuropathy, leading to delayed recognition and treatment [1-3]. Certain features may help distinguish POEMS from these mimickers, including prominent systemic findings (organomegaly, endocrine dysfunction, skin hyperpigmentation, edema), thrombocytosis or polycythemia, and the presence of sclerotic bone lesions or a lambda-restricted monoclonal plasma cell clone [1-5].

Epidemiologically, POEMS syndrome predominantly affects men in their fifth to sixth decade of life, with an estimated prevalence of around 0.3 per 100,000, although the true incidence is likely underestimated due to under-recognition [3,5]. Most available knowledge comes from case reports and retrospective series, with fewer than 1,000 thoroughly documented cases worldwide, underscoring the rarity of the condition [2,4,5]. In the absence of large randomized trials, management is guided by expert consensus and institutional experience, typically stratified by disease extent into localized therapy (radiotherapy) or systemic treatment with chemotherapy and autologous stem cell transplantation [5,6]. Prompt identification is essential, as targeted therapy can substantially improve both neurological recovery and overall prognosis [5,6].

The present case of a middle-aged male presenting with a CIDP-like neuropathy, newly diagnosed diabetes, thrombocytosis, and subtle skin changes illustrates how POEMS syndrome can initially mimic more common neuropathic disorders and how a systematic, multidisciplinary evaluation was required to uncover an underlying femoral plasmacytoma and establish the definitive diagnosis

## Case presentation

History

A 45-year-old male with hypertension, well controlled on perindopril and amlodipine, presented with a three-month history of progressive bilateral foot numbness and distal weakness, which had worsened over the past four weeks to involve proximal muscles, causing difficulty in walking. He denied any upper-limb involvement, back pain, or bladder and bowel disturbances, including urinary or fecal incontinence. There was no history of diplopia, dysarthria, dysphagia, fasciculations, seizures, or cognitive changes. He also denied fever, recent infections, falls, or travel. He reported intentional weight loss through dietary modifications. His past medical history was otherwise unremarkable, with no family history of neurological diseases. He was a non-smoker, had quit alcohol one year earlier, and followed a balanced diet without vegetarian restrictions.

Examination

On examination, the patient was comfortable lying in bed, alert, and oriented, with a Glasgow Coma Scale score of 15/15. Vital signs were stable, with a temperature of 36.6°C, heart rate of 75 beats per minute, respiratory rate of 18 breaths per minute, blood pressure at 142/75 mmHg, and oxygen saturation of 100% on room air.

Neurological examination revealed intact cranial nerves. The upper limbs showed normal tone, bulk, power (5/5), reflexes, and sensation. In the lower limbs, there was mild weakness (4/5) at the hips and knees, but severe weakness (0-1/5) at the ankles bilaterally, accompanied by hypotonia. Deep tendon reflexes were absent in both lower limbs, and plantar responses were absent bilaterally. Sensory examination demonstrated reduced sensation in a stocking distribution up to the ankles, with loss of light touch, pressure, joint position sense, and vibration up to the knees. Gait assessment revealed a high-steppage gait and a positive Romberg sign. No tongue or limb fasciculations were noted.

**Figure 1:**
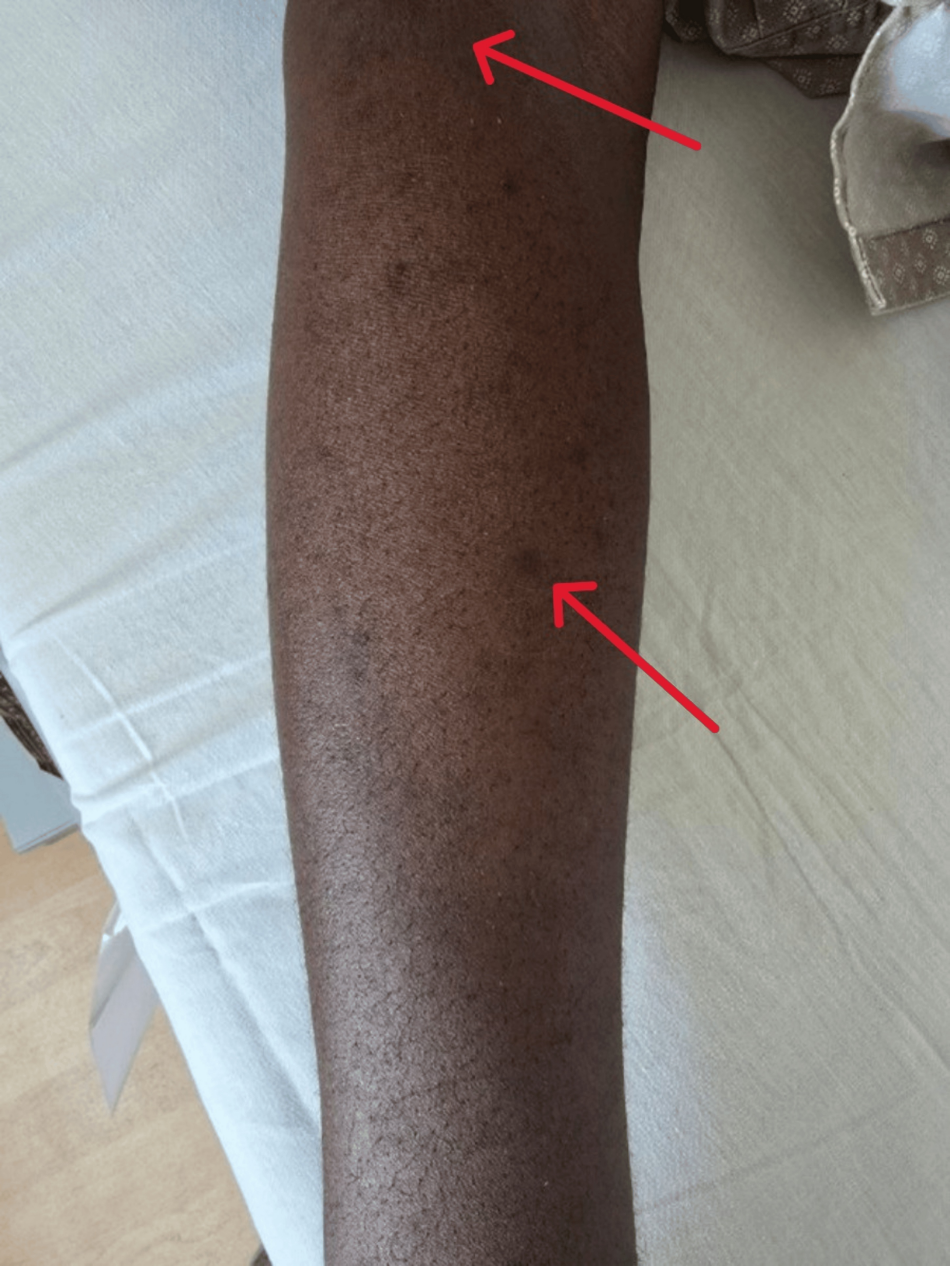
Skin discoloration consistent with hyperpigmentation over the right leg (red arrows).

Skin examination showed hyperpigmentation over the extremities (Figure 1). Cardiovascular examination revealed normal heart sounds without murmurs. Chest auscultation was clear with normal vesicular breath sounds, and the abdomen was soft, non-tender, and non-distended.

Investigation, management, and outcome

The patient was provisionally diagnosed with progressive distal and lower-limb-predominant sensorimotor peripheral neuropathy, with a possible diagnosis of CIDP, for further evaluation. Initial laboratory investigations revealed newly diagnosed diabetes mellitus with elevated glycated hemoglobin (HbA1c), thrombocytosis, and otherwise normal routine parameters, including hemoglobin and total protein levels. Mild coagulopathy was noted. Vitamin B12, folic acid, and thyroid function tests were within normal limits (Table 1).

**Table 1:** Laboratory investigations. CRP, C-reactive protein; PH, potential of hydrogen; ALT, alanine aminotransferase; AST, aspartate aminotransferase; HbA1c, glycated hemoglobin; TSH, thyroid-stimulating hormone; FT4, free thyroxine; PT, prothrombin time; INR, international normalized ratio; APTT, activated partial thromboplastin time.

Parameters	On admission	On discharge	Reference values
Platelet (x10^3/uL)	616	564	150-410 x10^3/uL
Total leukocytes	8.2	6	6.2 x10^3/uL
Hematocrit	45.9	41.9	40-50%
Hemoglobin (gm/dL)	15.7	14.5	13-17 gm/dL
Mean corpuscular volume (MCV) (fL)	90.2	91.4	83-101 fL
Mean corpuscular hemoglobin (MCH) (pg)	30.8	31.7	27-32 pg
CRP mg/L	11.2	<2	0-5 mg/L
Serum urea (mmol/L)	3.3	5	2.5-7.8
Serum creatinine (umol/L)	78	72	62-106
Serum potassium (mmol/L)	5.3	4.7	3.5-5.3
Serum sodium (mmol/L)	136	140	133-146
Serum calcium (mmol/L)	2.36	2.24	2.2-2.6
Serum phosphorus (mmol/L)	1.28	Not done	0.8-1.5 mmol/L
Serum magnesium (mmol/L)	0.84	Not done	0.7-1 mmol/L
PH	7.38	Not done	7.320-7.420
Serum total protein (gm/L)	72	74	60-80
Serum albumin (gm/L)	32	35	35-50
ALT (IU/L)	18	27	0-41
AST (IU/L)	15	18	0-41
Alkaline phosphatase (U/L)	95	98	40-129
Serum total bilirubin (mg/dl)	7	6	0-21
HbA1c	6.7	-	<6%
Random blood sugar level mmol/L	15.5	9.8	3.3-7.8 mmol/L
TSH (mIU/L)	2.18	Not done	0.34-4.20 mIU/L
FT4 (pmol/L)	16.1	Not done	11-23.3 pmol/L
PT (seconds)	14.8	13.5	9.4-12.5 seconds
INR	1.3	1.3	<1
APTT (seconds)	28.8	29.8	25.1- 36.5 seconds

Cerebrospinal fluid analysis revealed clear fluid with two cells (normal: 0-5 cells/µL), elevated total protein (0.64 g/L; reference range: 0.15-0.45 g/L), markedly increased immunoglobulin G (73 mg/dL; reference range: 0-4 mg/dL), slightly elevated albumin (357 mg/L; reference range: 100-350 mg/L), and negative oligoclonal bands (normal: negative).

Spinal MRI showed no abnormalities. Additional advanced investigations, including an antinuclear antibody (ANA) profile (Table 2), hepatitis B surface antigen, surface antibody, and core antibody, along with a positive PCR, indicated a newly diagnosed active hepatitis B infection.

**Table 2:** Summary of immunological work-up and renal biomarkers. β2M, beta-2 microglobulin; ANA, antinuclear antibody; dsDNA, double-stranded DNA; Ro52, Ro52 antigen; SS-A, Sjögren syndrome A; SS-B, Sjögren syndrome B; Sm, Smith antigen; RNP, ribonucleoprotein; PCNA, proliferating cell nuclear antigen; AMA-M2, anti-mitochondrial antibody M2; Jo-1, histidyl-tRNA synthetase; PM-Scl, polymyositis-scleroderma antigen.

Parameters	On admission	On discharge	Reference values
β2-microglobulin (mg/L)	2.45	Not done	(1.5 to 3 mg/L)
ANA profile (includes anti-dsDNA, anti-RO52, anti-SS-A, anti-nucleosomes, anti-Sm, anti-RNP, anti-histones, anti-PCNA, anti-SS-B, anti-ribosomal-P-protein, anti-JO1, anti-AMA-M2, anti-centromere B, anti-PM-Scl antibodies)	Negative	-	Negative

**Figure 2:**
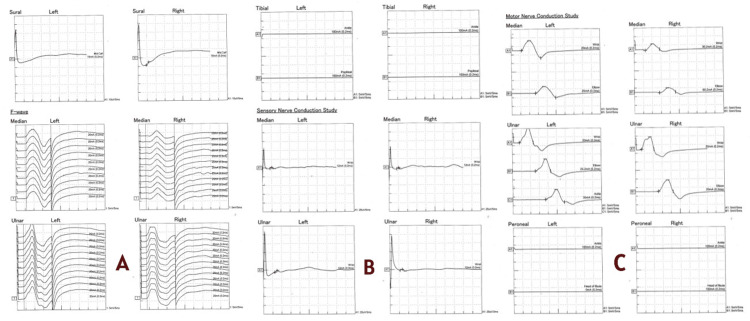
Nerve conduction studies (NCS) demonstrating abnormalities in both sensory and motor nerves. Panel A illustrates upper limb motor and sensory NCS recordings of the median and ulnar nerves bilaterally, demonstrating reduced compound muscle action potential (CMAP) amplitudes, prolonged distal latencies, and slowed conduction velocities, consistent with mixed demyelinating and axonal involvement. Panel B shows sensory NCS recordings of the median, ulnar, and sural nerves, with markedly reduced or attenuated sensory nerve action potentials (SNAPs) bilaterally, indicating significant sensory axonal loss. Panel C depicts lower limb motor NCS recordings of the tibial and peroneal nerves, revealing absent or severely reduced responses bilaterally, reflecting severe axonal involvement.

The neurology team was involved from the time of admission. A provisional diagnosis of CIDP was made, and treatment with intravenous methylprednisolone 1 g daily was initiated, later transitioned to oral prednisolone at 1 mg/kg/day. Despite a full course of intravenous methylprednisolone, followed by high-dose oral prednisolone, there was no clinically meaningful improvement in muscle strength or gait, which further argued against typical CIDP and prompted re-evaluation for alternative diagnoses, including POEMS syndrome. However, as the symptoms were predominantly distal in the lower limbs and associated with hyperpigmentation, the possibility of POEMS syndrome and paraproteinemic neuropathy was considered. Figure 2 depicts nerve conduction studies (NCS), including both sensory and motor nerve assessments. Panels A, B, and C demonstrate NCS of the upper and lower limb nerves, showing a bilateral mixed demyelinating and severe axonal polyradiculopathy.

**Figure 3:**
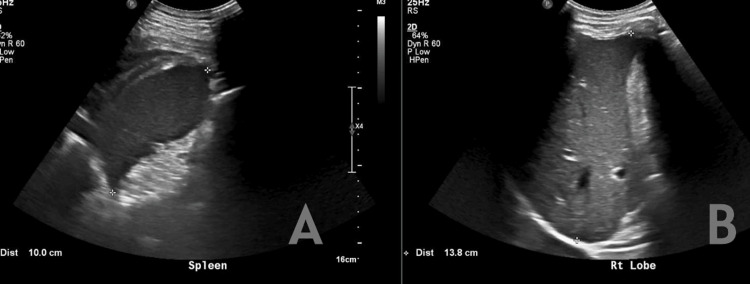
Abdominal ultrasound revealed normal liver and spleen spans with no hepatosplenomegaly. Panel A shows longitudinal ultrasound imaging of the spleen, demonstrating a normal splenic span (approximately 10.0 cm) with homogeneous echotexture and no evidence of splenomegaly. Panel B illustrates longitudinal ultrasound imaging of the right hepatic lobe, revealing a normal liver span (approximately 13.8 cm) with preserved parenchymal echogenicity and no focal lesions.

Abdominal ultrasound was unremarkable, with no evidence of organomegaly (Figure 3).

Serum protein electrophoresis showed two faint IgA lambda bands in the beta-gamma region, with positive CD138 and lambda staining. Hematology consultation was sought, and repeat serum protein electrophoresis, along with a PET scan, was recommended. The repeat electrophoresis confirmed the initial findings.

**Figure 4:**
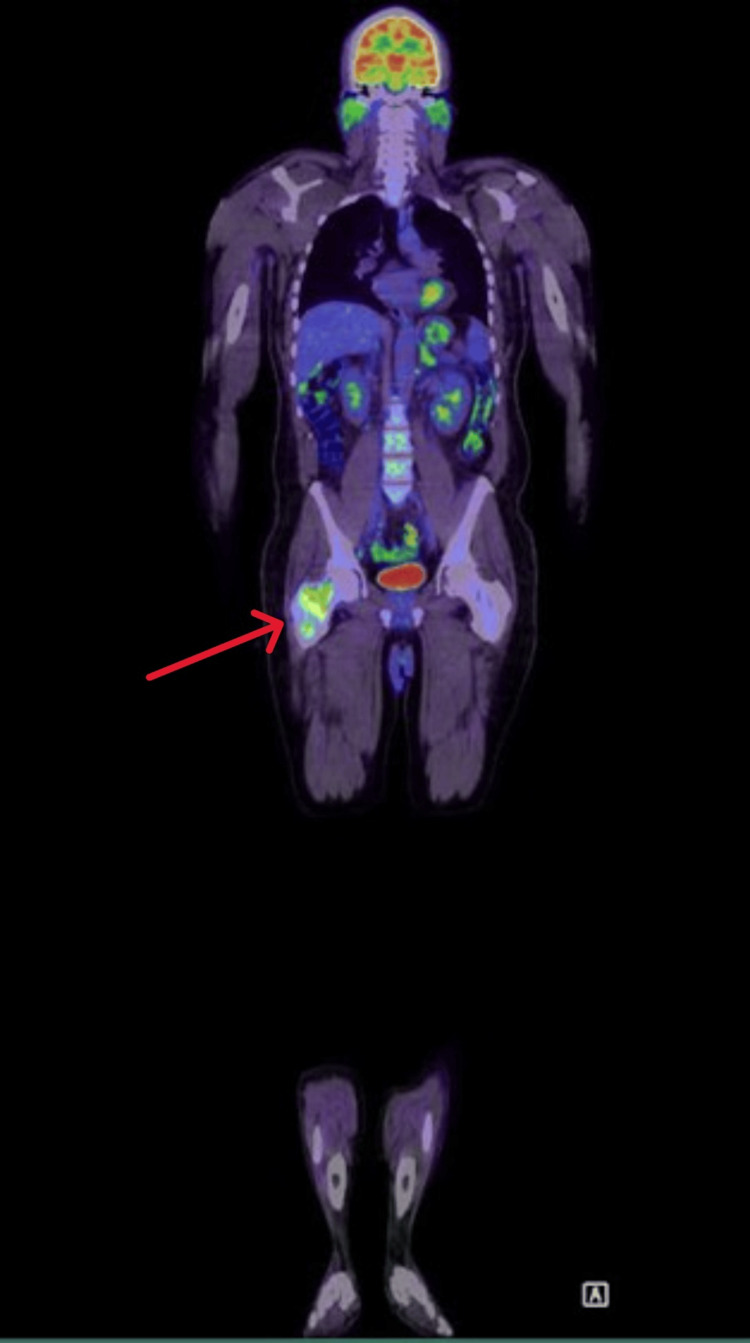
PET scan revealed a solitary destructive lesion in the neck of the right femur showing marked increased metabolic activity (red arrow).

A fluorodeoxyglucose PET scan demonstrated a solitary destructive lesion in the neck of the right femur with markedly increased metabolic activity (Figure 4), as well as a fat-poor left adrenal adenoma. Based on these findings, the case was discussed with the orthopedic team, who proceeded with a bone biopsy of the femoral lesion.

**Figure 5:**
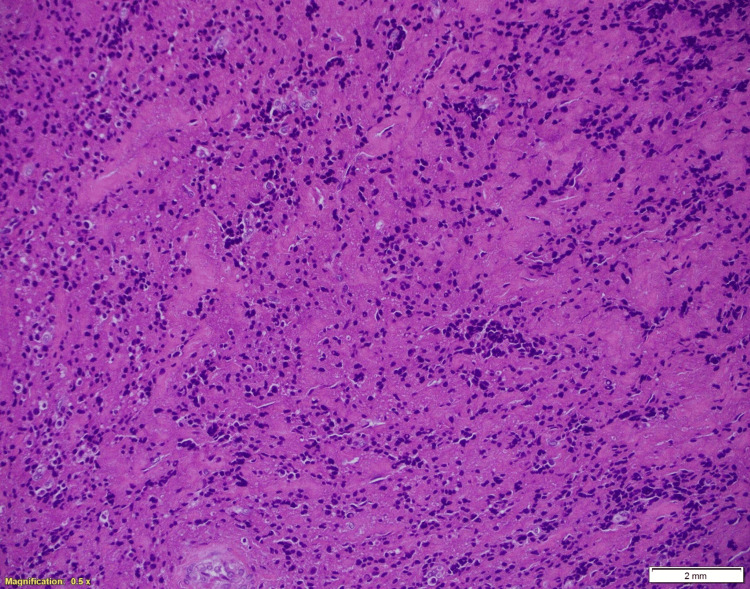
Bone biopsy (hematoxylin & eosin stain, x20) showing a nodular lesion within the bone marrow composed of sheets of crushed atypical plasma cells.

Histopathological analysis revealed bony fragments infiltrated by sheets of lambda-restricted plasma cells, consistent with a plasma cell neoplasm (plasmacytoma) (Figure 5).

**Figure 6:**
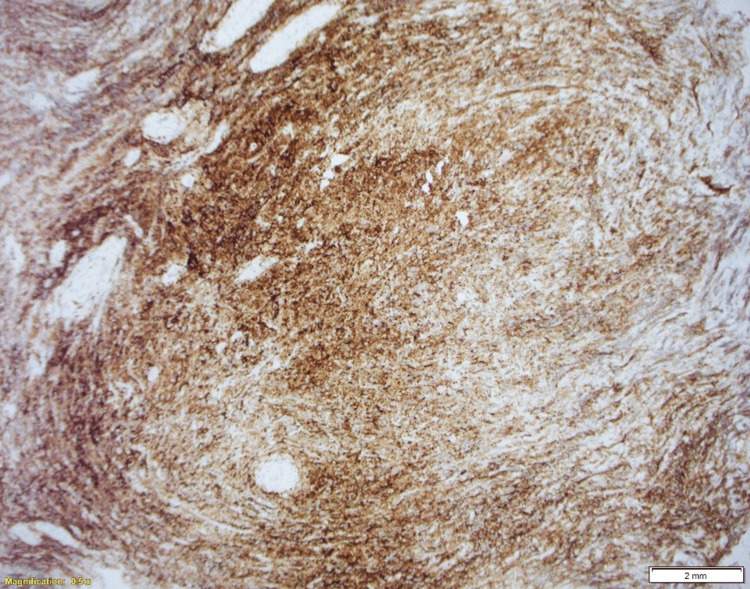
CD138 immunostain confirms plasma cells.

**Figure 7:**
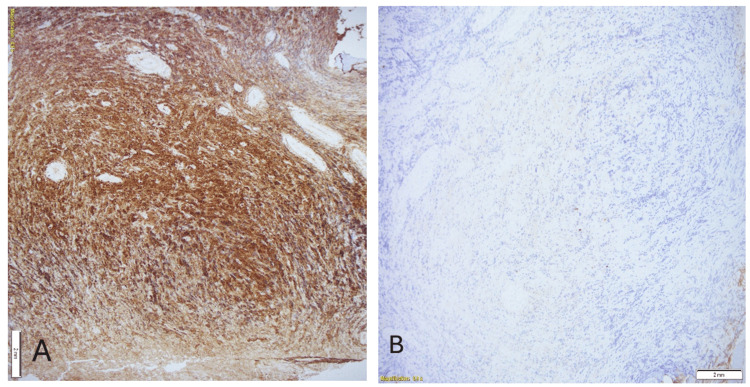
(A, B) Lambda and kappa. Lambda-restricted pattern of staining.

Immunohistochemistry was positive for CD138 (Figure 6) and lambda (Figure 7), and negative for kappa, CD45, CD3, CD20, CKAE1/3, CD99, and synaptophysin. Bone marrow aspiration and biopsy showed aberrant CD56 expression and lambda light-chain predominance on flow cytometry.

Follow-up

Following multidisciplinary discussions among the neurology, hematology, and internal medicine teams, a final diagnosis of POEMS syndrome was established based on accepted diagnostic criteria, including polyneuropathy, a monoclonal lambda-restricted plasma cell disorder, plasmacytoma, thrombocytosis, and skin hyperpigmentation. The patient was subsequently referred to the hematology, hepatology, oncology (for planned targeted radiotherapy), physiotherapy, and neurology services for ongoing outpatient follow-up and management. He was initiated on external-beam radiotherapy (XRT) for the femoral plasmacytoma under the care of the radiation oncology team and remains on regular multidisciplinary follow‑up with hematology, neurology, and radiation oncology. At the time of last review, he was still undergoing treatment and had demonstrated modest functional gains, with partial improvement in distal lower‑limb strength and gait stability, although significant residual neuropathic deficits persisted. Ongoing management focuses on completion of local radiotherapy, physiotherapy‑guided rehabilitation, and longitudinal hematologic and neurological surveillance to monitor for disease control and further neurological recovery.

## Discussion

This patient’s presentation fulfilled the diagnostic criteria for POEMS syndrome, demonstrating the two mandatory major features (polyneuropathy and a monoclonal plasma cell disorder), along with an additional primary criterion of a bone lesion. Minor features included thrombocytosis and skin hyperpigmentation. Although vascular endothelial growth factor (VEGF) levels were not assessed, the overall clinical and pathological profile strongly supported the diagnosis. Limited plasma cell infiltration in the bone marrow aligns with typical POEMS pathology, in which isolated plasmacytomas are more common than diffuse marrow involvement [4]. Histopathology from the femoral lesion revealed sheets of monoclonal plasma cells positive for CD138 and lambda light chain, confirming plasma cell lineage and light-chain restriction. Recognition of this constellation of findings is essential, as early diagnosis enables targeted therapy that addresses both the plasma cell neoplasm and its systemic manifestations, ultimately improving patient outcomes [1,5].

POEMS syndrome is a rare multisystem disorder defined by the combination of peripheral neuropathy and a plasma cell dyscrasia, with additional features such as organomegaly, endocrinopathies, and skin changes. It has been historically referred to as osteosclerotic myeloma, Crow-Fukase syndrome, or PEP (polyneuropathy-endocrinopathy-plasma cell dyscrasia) syndrome [2]. The diagnostic framework, established by Dispenzieri et al., requires both mandatory major criteria, polyneuropathy, and a monoclonal plasma cell disorder, plus at least one other major and one minor criterion [1,6]. Because of its heterogeneous presentation, POEMS syndrome is frequently misdiagnosed as CIDP or other neuropathies [3]. It typically affects patients in their fifth to sixth decade of life, with a slight male predominance and a prevalence of approximately 0.3 per 100,000 [3,5].

Most available data on POEMS syndrome arise from case reports and retrospective case series rather than randomized clinical trials, reflecting its rarity. Since its initial description, fewer than 1,000 cases have been comprehensively documented worldwide [5,6]. Significant contributions come from large series published by the Mayo Clinic and Japanese cohorts, which helped refine the diagnostic criteria and treatment recommendations [5,6]. Despite advances, the lack of randomized evidence means that management remains guided mainly by expert consensus and institutional experience, emphasizing the importance of multidisciplinary collaboration [1,5].

The pathophysiology of POEMS syndrome is multifactorial but strongly linked to overproduction of proinflammatory and angiogenic cytokines, particularly VEGF, which increases vascular permeability and contributes to systemic manifestations such as edema, effusions, and organomegaly [1,5,6]. However, despite the strong association, anti-VEGF therapies have shown limited success, suggesting VEGF acts as a biomarker rather than the sole pathogenic driver [6]. Other cytokines, including interleukin-1β, tumor necrosis factor-alpha (TNF-α), IL-6, and IL-12, are frequently elevated and may mediate systemic inflammation and vascular damage [7]. Notably, interleukin-6 has been identified as a highly prognostic biomarker for disease activity and outcomes [8]. The inflammatory milieu is further supported by elevated levels of matrix metalloproteinases and tissue inhibitors, such as TIMP-1 [9].

Clinically, the disease progresses insidiously, with length-dependent, symmetric peripheral neuropathy as the predominant feature in all patients [1,9]. The neuropathy is typically demyelinating, sensorimotor, and progressive, often leading to distal weakness, sensory loss, and foot drop. In most cases, the paraprotein is lambda-restricted, as observed in our patient [1]. Other major diagnostic features include osteosclerotic bone lesions, elevated VEGF, or Castleman disease, particularly the human herpesvirus-8 (HHV-8)-negative multicentric type [10]. Minor criteria commonly include endocrinopathy, skin hyperpigmentation, organomegaly, extravascular volume overload, and hematologic abnormalities such as thrombocytosis or polycythemia [1,2].

There is no universally accepted standard therapy for POEMS syndrome, and management is tailored to the extent of the disease. In patients with limited disease (defined as one to three isolated sclerotic lesions without diffuse marrow involvement), targeted radiotherapy is preferred, as it can be curative with minimal toxicity and preserves systemic treatment options if progression occurs [11,12]. For patients with disseminated or systemic disease, therapy generally mirrors that used in multiple myeloma. Lenalidomide-based regimens are preferred due to their lower neurotoxicity than bortezomib [13,14]. High-dose melphalan followed by autologous stem cell transplantation (ASCT) remains the standard of care for transplant-eligible patients, offering durable hematologic and clinical responses [13]. For those ineligible for ASCT, combinations of lenalidomide or thalidomide with corticosteroids remain effective alternatives, although thromboprophylaxis is essential due to increased thrombotic risk [14]. Treatment response is monitored via hematologic parameters, imaging, and clinical improvement; neurological recovery is typically slow, requiring months to stabilize and years for maximal improvement [14].

If left untreated, POEMS syndrome is progressive and potentially fatal, leading to worsening neuropathy, ascites, pulmonary hypertension, and multiorgan dysfunction. However, advances in diagnosis and therapy have dramatically improved survival, with 10-year survival rates approaching 80% in recent studies [15,16]. Favorable prognostic indicators include younger age, higher serum albumin levels, and achievement of a complete hematologic response [15,16]. Conversely, the presence of pleural effusions, renal impairment, pulmonary hypertension, or coexisting Castleman disease is associated with poorer outcomes [15,16].

Overall, this case reinforces the importance of maintaining a high index of suspicion for POEMS syndrome in patients presenting with unexplained progressive neuropathy and systemic signs. Early diagnosis, multidisciplinary coordination, and targeted therapy directed at the plasma cell clone remain the cornerstones of optimal management, improving both survival and neurological recovery [1,5,14].

## Conclusions

This case highlights the importance of maintaining a high index of suspicion for POEMS syndrome in patients presenting with progressive, initially CIDP‑like peripheral neuropathy accompanied by systemic manifestations such as thrombocytosis, skin hyperpigmentation, newly diagnosed diabetes, and an underlying femoral plasmacytoma, even when routine investigations are initially compatible with CIDP.
